# Pregnancy and Neonatal Outcomes in SARS-CoV-2 Infection: A Systematic Review

**DOI:** 10.1155/2020/4592450

**Published:** 2020-10-07

**Authors:** Reem S. Chamseddine, Farah Wahbeh, Frank Chervenak, Laurent J. Salomon, Baderledeen Ahmed, Arash Rafii

**Affiliations:** ^1^Genetic Intelligence Laboratory, Weill Cornell Medicine-Qatar, Doha, Qatar; ^2^Department of Obstetrics and Gynecology, Zucker School of Medicine at Hofstra/Northwell-Lenox Hill Hospital, New York, USA; ^3^Maternité, Médecine, Chirurgie et Imagerie Fœtales, Hôpital Universitaire Necker Enfants Malades, Université de Paris and EA FETUS, Affiliate of Institute Imagine, Paris, France; ^4^Fetal Maternal Center, Doha, Qatar; ^5^Qatar University, Doha, Qatar; ^6^Department of Genetic Medicine and Obstetrics and Gynecology, Weill Cornell Medicine, New York, USA

## Abstract

With the emergence of SARS-CoV-2 and its rapid spread, concerns regarding its effects on pregnancy outcomes have been growing. We reviewed 245 pregnancies complicated by maternal SARS-CoV-2 infection across 48 studies listed on PubMed and MedRxiv. The most common clinical presentations were fever (55.9%), cough (36.3%), fatigue (11.4%), and dyspnea (12.7%). Only 4.1% of patients developed respiratory distress. Of all patients, 89.0% delivered via cesarean section (*n* = 201), with a 33.3% rate of gestational complications, a 35.3% rate of preterm delivery, and a concerning 2.5% rate of stillbirth delivery or neonatal death. Among those tested, 6.45% of newborns were reported positive for SARS-CoV-2 infection. Relative to known viral infections, the prognosis for pregnant women with SARS-CoV-2 is good, even in the absence of specific antiviral treatment. However, neonates and acute patients, especially those with gestational or preexisting comorbidities, must be actively managed to prevent the severe outcomes being increasingly reported in the literature.

## 1. Introduction

The emergence of SARS-CoV-2 as a novel infection in late December 2019 poses unique challenges to healthcare systems and practitioners. Chief among them is the management for pregnant women who are infected with SARS-CoV-2. Pregnant women are prone to a range of fetal and maternal complications that could impact the outcome of any concurrent infection [1]. The first trimester bears a major risk for miscarriage and fetal developmental abnormalities. The late second and third trimesters carry an increased likelihood for the development of maternal conditions, such as gestational diabetes and hypertensive disorders, which contribute to maternal morbidity and premature birth.

Cells at the maternal-fetal interface play crucial roles in fetal development and regulation of the maternal immune response [2]. Viral infections can interfere with the function of these cells and ultimately impair placental function [2]. Over the years, many studies have investigated the maternal and neonatal outcomes in the setting of several recent epidemics such as influenza A (H1N1), SARS-CoV, Middle East Respiratory Syndrome (MERS), and Respiratory Syncytial Virus (RSV). A case-control study (deaths and recovered) conducted in Brazil on women infected with influenza A (H1N1) reported a higher rate of fetal deaths and preterm births among women who eventually died as a result of the infection, compared to women who had recovered. Further, recovered cases had more favorable neonatal outcomes following discharge [3]. The authors reported that early antiviral treatment (48 to 72 hours of symptom onset) is a protective factor, whereas having a previous visit to a healthcare provider for an influenza episode prior to hospital admission was a risk factor for death [3]. A systematic review conducted in 2017 on maternal influenza infection reported preterm birth rates (prior to 37 weeks of gestation) of 11.4%, 7.7%, 7.1%, and 6.3% in the United States, Canada, the United Kingdom, and Norway, respectively [4]. In pregnancies affected by respiratory syncytial virus (RSV), the rate of preterm births was reported as 29%, which is comparable to a rate of 13% for preterm births in noninfected pregnant women [5]. Among infants born to mothers with postpartum RSV infection, 57% were infected with RSV [5]. A systematic review published in 2020 compared maternal and neonatal outcomes among patients infected with either SARS-CoV, SARS-CoV-2, or MERS. When compared to SARS-CoV and MERS, SARS-CoV-2 was associated with the highest rate of preterm birth, fetal distress, perinatal death, and C-section deliveries [6]. Additionally, no Apgar scores < 7 were reported in any of the SARS-CoV and MERS cases, whereas 2.4% of SARS-CoV-2 cases had Apgar scores < 7 [6]. There was no evidence of vertical transmission in any of the three infections [6].

It is well known that infectious pneumonia is a common cause of morbidity and mortality in pregnant women due to several physiological factors such as lower lung volumes and increased oxygen consumption [7, 8]. Indeed, a quarter of pregnancies complicated by pneumonia are estimated to require critical care hospitalization and ventilation support [9]. Pregnancy-induced changes to immunity, such as reduction in cell-mediated cytotoxicity, diminished lymphokine response, and reduction in lymphocyte proliferative response, further add to the prognostic picture of pneumonia in pregnant women [8]. Hence, the advent of the SARS-CoV-2 pandemic could raise new questions about perinatal and obstetric management. Here, we review 245 pregnancies reported in the literature across 48 studies.

## 2. Methods

**Figure 1:**
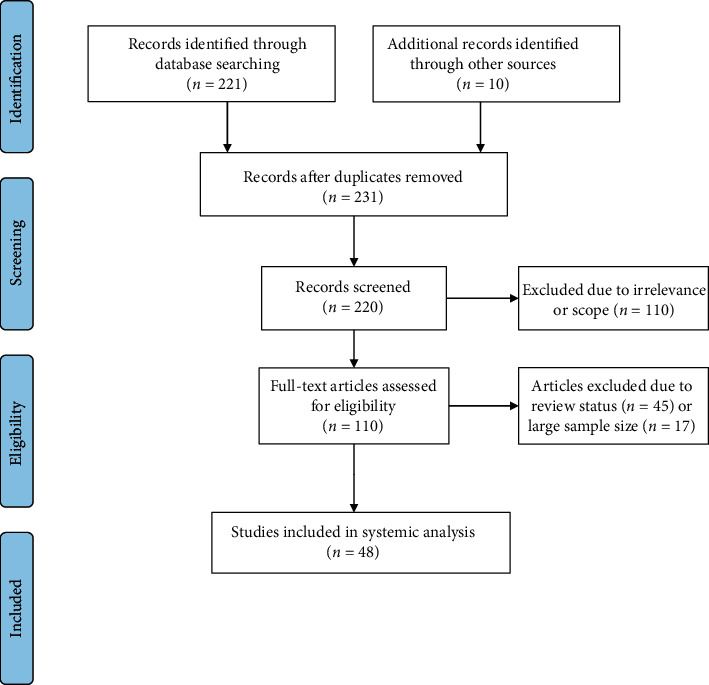
Search strategy flowchart.

We searched PubMed using the All Fields and MeSH tags for the terms “coronavirus,” “ncov,” “cov,” “2019-nCoV,” “COVID-19,” “SARS-CoV-2,” “pregnancy,” “complications,” “miscarriage,” and “anomalies.” We limited our search to all published studies between December 20, 2019, and July 30, 2020. Our search retrieved 221 papers. We carried out the same search on MedRxiv, which yielded 10 results. We screened the papers through Covidence. We excluded reviews, meta-reviews, letters to the editor, and guidelines that are specific to a region. We also excluded articles in languages besides English. We then reviewed the results for relevance and included the articles that reported on SARS-CoV-2 in pregnant patients. This search strategy resulted in 48 case reports and articles from the primary literature that we included in this review (Figure 1). We extracted all information about patient characteristics, symptoms, laboratory results, and imaging studies. We also gathered the relevant data about the pregnancy course such as delivery method, gestational complications, and neonatal outcomes. The data from each article were recorded at the level of individual patients and combined across studies for calculating the frequency of the outcomes in all patients.

## 3. Results

The papers we reviewed consist of 245 patients, all of whom are pregnant women with a confirmed diagnosis of SARS-CoV-2, with a mean maternal age of 28.3 (range of 24-43 years old, *n* = 241) and a mean gestational age (GA) of 35.67 weeks (range 18-41 weeks, *n* = 119). Results from individual studies are described and summarized in Supplementay Tables 1 and 2.

### 3.1. Maternal Symptoms

With regard to prepartum symptoms, the most common symptoms were fever (55.9%) followed by cough (36.3%), dyspnea (12.7%), and fatigue (11.4%). Less common symptoms included diarrhea and other gastrointestinal (GI) symptoms, sore throat, nasal congestion and rhinorrhea, muscle aches, rash, headache, hoarseness, myalgia, high blood pressure (BP), tachycardia, and cholecystitis. Only 4.5% of all patients were asymptomatic prior to delivery. Several patients who were asymptomatic prior to delivery became symptomatic postpartum, requiring ICU admission.

The majority of the studies only included patients in the third trimester with the exception of eight studies which also included patients in the first and second trimesters. Most of these studies reported the number of patients experiencing each symptom without specifying the GA, so it is difficult to determine whether patients in earlier trimesters differed in their clinical presentations from those in later trimesters. Liu and colleagues reported 15 patients who ranged in GA from 12 to 38 weeks, but it was not clear whether the patients in the earlier trimester experienced different symptoms or complications [10]. Nie included 33 patients in total which ranged in GA from 24 to 36 weeks [11]. No ICU admissions or mortalities were reported, and the study concluded that pregnant women were not at an increased risk for severe illness or mortality. Based on the findings reported by Liu et al. and Nie, patients' clinical presentations do not appear to differ based on the GA of pregnancy.

Among the reported postpartum symptoms, the most common one was fever (13.5%). In one patient who was admitted with worsening hypertension and a history of diabetes mellitus (DM) and asthma, postpartum symptoms included low oxygen saturation, tachycardia, dyspnea, diaphoresis, and cough that progressed to respiratory failure [12]. Gidlöf et al. also reported low-oxygen saturation postpartum [13]. Among 13 patients, Liu and colleagues reported that one patient developed Multi Organ Dysfunction Syndrome (MODS) including Acute Respiratory Distress Syndrome (ARDS) [14]. Thus, out of 245 pregnant women with SARS-CoV-2, 10 (4.1%) developed respiratory distress.

### 3.2. Laboratory Findings

The most common laboratory abnormalities seen in pregnant women infected with SARS-CoV-2 were elevated C-reactive protein (CRP), neutrophilia, and leukocytosis. In the studies that reported laboratory findings, 147 pregnant women were included, and among them, 49% had elevated CRP, 31.3% had lymphopenia, 28.6% had neutrophilia, and 12.2% had leukocytosis. Other laboratory abnormalities included thrombocytopenia, elevated D-dimer, abnormal liver enzymes, decreased albumin, low hemoglobin (Hb), elevated alkaline phosphate, eosinopenia, elevated uric acid, elevated ferritin, pancytopenia, prolonged activated partial thromboplastin time (APTT), low fibrinogen, and progressive coagulopathy.

### 3.3. Chest X-Ray (CXR)

Most studies reported imaging abnormalities in either chest X-ray (CXR), computed tomography (CT), or both. In terms of CXR, Li et al. (*n* = 1) reported scattered multiple patchy infiltrates in both lungs, and Lee et al. (*n* = 1) reported left lower/middle lobe consolidation and increased vascular marking, while Breslin et al. (*n* = 1) reported mild pulmonary vascular congestion with no consolidation or effusion [12, 15, 16]. All three women were of similar GA (mean 36 weeks).

### 3.4. Computed Tomography (CT) Scan

Typical signs of viral pneumonia were frequently reported, with most patients having bilateral lung involvement. CT findings did not show signs of worsening pneumonia postpartum. However, with disease progression, Zhu et al. reported that lesions merged into strips and Liu et al. observed a paving pattern and consolidation [10, 17]. Ground glass opacities (GGO) were reported in the majority of studies. Mixed GGO with consolidation were more common among pregnant SARS-CoV-2 patients in comparison to nonpregnant SARS-CoV-2 patients [10]. Lee and colleagues also reported GGO with consolidation [15]. In addition to multiple bilateral consolidations, bilateral pleural effusion were observed [18]. Given the prevalence of respiratory symptoms in pregnant women infected with SARS-CoV-2, lung ultrasound is a valuable tool in confirming diagnosis of SARS-CoV-2 infection as well as monitoring disease progression [19]. Lung ultrasound eliminates the radiation risk and avoids the incidence of false-negative results associated with other diagnostic tests. As outlined by Moro and colleagues, specific guidelines can be followed when performing lung ultrasound for pregnant women infected with SARS-CoV-2 [19].

### 3.5. Maternal and Obstetric Complications

There have been several reports of uncomplicated term delivery in SARS-CoV-2-positive women [15, 20]. Of 201 deliveries reported, 182 were performed as C-sections. The rate of C-sections is thus 89% of all deliveries, significantly higher than the expected rate of approximately 15% for pregnancies in the general population [21, 22]. This rate of C-section is also higher than that in pregnancies complicated by viral Influenza infection, where the likelihood of C-section did not increase relative to the general population [23].

Several case reports highlight the frequency of C-section deliveries compared to vaginal deliveries, whether in healthy-appearing patients or patients presenting with fever [17, 24]. The indications for C-section varied and were not consistently reported across studies. In certain hospitals, the choice of C-section was determined by obstetric factors [25]. In others, C-section was performed proactively due to maternal SARS-CoV-2 infection. Presumably, C-sections minimize cross-infection and reduce maternal exertion during labor, as per certain recommendations [26]. Due to the uneven distribution of C-sections as opposed to vaginal delivery, it is not possible to comment on differences in neonatal and maternal outcomes between vaginal delivery and C-sections in SARS-CoV-2 pregnancies. Importantly, there are no contraindications for vaginal delivery as evidenced by the uncomplicated vaginal delivery in at least five patients [27, 28]. It is not clear from this review if the high cesarean section rate is warranted.

Out of 245 patients reviewed, we looked at the following gestational complications: gestational diabetes, preeclampsia/gestational hypertension, and placental complications, such as premature rupture of membrane or placenta previa. The combined rate of complications was at 33.3%, consisting of gestational diabetes (6.9%), preeclampsia/gestational hypertension (6.1%), and placental complications (6.5%). There were at least 34 patients (13.8%) with other overlapping complications, including 9 cases of intrauterine fetal demise in the second and third trimesters (3.6%), 8 cases of maternal mortality (3.2%), and 7 cases of maternal hypoxia (2.8%). It is worth noting that 7 out of the eight cases of maternal mortality occurred among patients within the same study in the same hospital, and thus, the rate of maternal mortality may seem falsely elevated [29].

### 3.6. Fetal and Neonatal Outcomes

The literature reviewed reported information for 201 newborns. Out of 201 neonates, 71 (35.3%) were delivered preterm, before 36 weeks of gestation. Again, it is not consistently clear whether early delivery was induced in light of obstetric indications or maternal SARS-CoV-2 infection. The average Apgar score was 6.49 at 1 minute and 8.98 at 5 minutes.

Seven fetuses (2.8%) exhibited fetal distress, which was the indication for preterm delivery in some cases. Postnatally, the rate of abnormal respiratory findings among the neonates was at 4.4%, divided as follows: 6 neonates had shortness of birth, 2 had pneumonia-like presentation on imaging, and one had respiratory distress including one case of ARDS. All cases resolved with antibiotics without complication.

Of the 201 fetuses, five (2.5%) had very poor outcomes: two deliveries resulted in stillbirth, and three neonates passed away soon after delivery [14, 17, 29, 30]. One neonate developed refractory shock and multiple organ failure and passed on day 8 of life, while the other two were delivered by a mother who developed hypoxia in late pregnancy [17, 29]. Although no fetus is thought to have died as a result of fetal infection, Baud et al. suggest that placental inflammation in the setting of the virus contributes to fetal distress, even when the fetal is not infected [30].

### 3.7. Vertical Transmission and Breastfeeding

Notably, several case studies have ruled out vertical transmission of SARS-CoV-2. This is largely supported by our review. Out of the neonates tested for SARS-CoV-2 infection (*n* = 93), 6 tested positive for the infection through nasopharyngeal swab (6.45%). All cases in question raise concerns about potential vertical transmission, since the mother and the obstetric team practiced protection and isolation measures to prevent infection during the delivery process and immediately afterwards [11, 18]. However, because of the asymptomatic presentation of many infected individuals, it is possible that the neonates had acquired SARS-Cov-2 in the hospital or in their home environment after birth.

Certain studies went beyond testing the newborn for evidence of vertical transmission. For instance, Gidlöf et al. tested breast milk and maternal vaginal secretion of a SARS-CoV-2-positive mother [13]. Li et al. tested the breast milk, amniotic fluid, umbilical cord blood and placenta, maternal serum, urine, and feces of a SARS-CoV-2-positive mother [14]. Five out of 7 placentas tested (71.4%) were positive for SARS-CoV-2, while 2 out of 13 tissue samples tested (15.3%) were positive.

One out of 6 breast milk samples tested (16.6%) was positive for SARS-CoV-2. Interestingly, SARS-CoV-2 IgG and IgA were detected in a placental tissue and breast milk sample, respectively [31]. These results, in addition to several guidelines such as those published by the ACOG and the RCOG, converge on the idea that SARS-CoV-2 is not carried through breast milk [32, 33]. However, the underlying risk of neonatal exposure to maternal respiratory droplets during the breastfeeding process persists. Overall, the many benefits of breastfeeding do make breastfeeding safe, as long as SARS-CoV-2-positive mothers take reasonable precautions while in close contact with the newborn.

## 4. Conclusions

According to Liu et al., pregnancy and delivery did not aggravate the severity of SARS-CoV-2 in the patients studied [10]. However, as one expects, the variability in the severity of infection and pneumonia presentation in different patients makes such a statement difficult to prove.

Pregnancy, in particular, the third trimester, is associated with changes in lung physiology, such as decreases in ERV and FRC volumes, and increases in respiratory resistance [34]. A prime example is the fact that pregnant women infected with the H1N1 virus during the 2009 epidemic were at a significantly higher risk of complications and hospitalization than nonpregnant women [35, 36]. Hence, an increased rate of respiratory complications, in addition to maternal and neonatal complications, could be expected in pregnant patients with SARS-CoV-2 infection. The absence of widespread respiratory complications is surprising and suggests that a larger number of patients will help better understand the natural history of SARS-CoV-2 infections during pregnancy.

It is certainly true that, at least from the available reports, the mortality risk in SARS-CoV-2 pregnant women is low. There exists only eight cases of maternal mortality and ten other cases of patients developing respiratory distress [12–14]. At the time of writing, there exists only reports of severe complications during late pregnancy and no reports of termination due to early congenital defects. In the 2002-2004 SARS epidemic, miscarriage rates were reported to be as high as 50% for infected women in the first trimester [37]. Similarly, infection with the seasonal influenza virus is associated with higher miscarriage and maternal mortality rates than the noninfected population [1, 35]. It should be noted however that miscarriage could be due to the maternal response to the infection, rather than a direct placental effect induced by the infection [38]. In the case of SARS-CoV-2, it is highly likely that the higher rate of stillbirths and IUFD cases could be attributed to fever. In contrast to these viral infections, the outcomes for SARS-CoV-2-infected pregnant women in the first trimester are reassuring.

With regard to fetal and neonatal outcomes, pneumonias during pregnancies are associated with an increased risk of preterm birth compared to the general population [39]. Our findings are consistent with this observation. Fortunately, premature neonates fared well and had comparable Apgar scores to the full-term neonates included in the analysis. Unfortunately, the rate of stillbirth and neonatal death among this group was 2.5%, significantly higher than the rate among the general population. Intrauterine growth restriction (IUGR) was an expected finding in this group due to its prevalence in prior SARS infections during pregnancy [40]. However, there was little reporting on IUGR among fetuses in SARS-CoV-2-positive pregnancies.

Although our review includes a high number of patients, it is limited by the heterogeneity of the studies' primary outcomes and reporting methods. Secondly, the availability of few reports of severe outcomes makes it difficult to explain the pathophysiology of stillbirths and maternal morbidity in the setting of SARS-CoV-2. Thirdly, the small sample scope of placental tissue and breast milk samples challenges the assumed null risk of transmission between the mother and the baby. As more case reports become available, subgroup analyses will enable stronger understanding of the impact of the virus on maternal and fetal health.

## Supplementary Materials

Supplementary Materials Table 1. Patient Characteristics and Symptoms in SARS-CoV-2 positive pregnancies. Table 2. Summary of Gestational Complications and Delivery Outcomes in SARS-CoV-2 positive pregnancies.

## References

[1] Mertz D., Geraci J., Winkup J., Gessner B. D., Ortiz J. R., Loeb M. (2017). Pregnancy as a risk factor for severe outcomes from influenza virus infection: a systematic review and meta-analysis of observational studies. *Vaccine*.

[2] Racicot K., Mor G. (2017). Risks associated with viral infections during pregnancy. *The Journal of Clinical Investigation*.

[3] Ribeiro A. F., Pellini A. C. G., Kitagawa B. Y. (2018). Severe influenza A(H1N1)pdm09 in pregnant women and neonatal outcomes, State of Sao Paulo, Brazil, 2009. *PLoS One*.

[4] Fell D. B., Savitz D. A., Kramer M. S. (2017). Maternal influenza and birth outcomes: systematic review of comparative studies. *BJOG: An International Journal of Obstetrics & Gynaecology*.

[5] Chu H. Y., Katz J., Tielsch J. (2016). Clinical presentation and birth outcomes associated with respiratory syncytial virus infection in pregnancy. *PLoS One*.

[6] Di Mascio D., Khalil A., Saccone G. (2020). Outcome of coronavirus spectrum infections (SARS, MERS, COVID-19) during pregnancy: a systematic review and meta-analysis. *American Journal of Obstetrics & Gynecology MFM*.

[7] Berkowitz K., LaSala A. (1990). Risk factors associated with the increasing prevalence of pneumonia during pregnancy. *American Journal of Obstetrics and Gynecology*.

[8] Brito V., Niederman M. S. (2011). Pneumonia complicating pregnancy. *Clinics in Chest Medicine*.

[9] Madinger N. E., Greenspoon J. S., Ellrodt A. G. (1989). Pneumonia during pregnancy: has modern technology improved maternal and fetal outcome?. *American Journal of Obstetrics and Gynecology*.

[10] Liu D., Li L., Wu X. (2020). Pregnancy and perinatal outcomes of women with coronavirus disease (COVID-19) pneumonia: a preliminary analysis. *American Journal of Roentgenology*.

[11] Nie R., Wang S.-s., Yang Q. Clinical features and the maternal and neonatal outcomes of pregnant women with coronavirus disease 2019.

[12] Breslin N., Baptiste C., Miller R. (2020). Coronavirus disease 2019 in pregnancy: early lessons. *American Journal of Obstetrics & Gynecology MFM*.

[13] Gidlöf S., Savchenko J., Brune T., Josefsson H. (2020). COVID-19 in pregnancy with comorbidities: more liberal testing strategy is needed. *Acta Obstetricia et Gynecologica Scandinavica*.

[14] Liu Y., Chen H., Tang K., Guo Y. (2020). Clinical manifestations and outcome of SARS-CoV-2 infection during pregnancy. *Journal of Infection*.

[15] Lee D. H., Lee J., Kim E., Woo K., Park H. Y., An J. (2020). Emergency cesarean section performed in a patient with confirmed severe acute respiratory syndrome coronavirus-2 -a case report-. *Korean Journal of Anesthesiology*.

[16] Li Y., Zhao R., Zheng S. (2020). Lack of vertical transmission of severe acute respiratory syndrome coronavirus 2, China. *Emerging Infectious Diseases*.

[17] Zhu H., Wang L., Fang C. (2020). Clinical analysis of 10 neonates born to mothers with 2019-nCoV pneumonia. *Translational Pediatrics*.

[18] Alzamora M. C., Paredes T., Caceres D., Webb C. M., Valdez L. M., La Rosa M. (2020). Severe COVID-19 during pregnancy and possible vertical transmission. *American Journal of Perinatology*.

[19] Moro F., Buonsenso D., Moruzzi M. C. (2020). How to perform lung ultrasound in pregnant women with suspected COVID-19. *Ultrasound in Obstetrics & Gynecology*.

[20] Yu N., Li W., Kang Q. (2020). Clinical features and obstetric and neonatal outcomes of pregnant patients with COVID-19 in Wuhan, China: a retrospective, single-centre, descriptive study. *The Lancet Infectious Diseases*.

[21] Librero J., Peiró S., Belda A., Calabuig J. (2014). Porcentaje de cesáreas en mujeres de bajo riesgo: un indicador útil para comparar hospitales que atienden partos con riesgos diferentes. *Revista Española de Salud Pública*.

[22] Betran A. P., Torloni M. R., Zhang J. (2015). What is the optimal rate of caesarean section at population level? A systematic review of ecologic studies. *Reproductive Health*.

[23] He J., Liu Z.-W., Lu Y.-P. (2017). A Systematic Review and Meta-Analysis of Influenza A Virus Infection During Pregnancy Associated with an Increased Risk for Stillbirth and Low Birth Weight. *Kidney and Blood Pressure Research*.

[24] Fan C., Lei D., Fang C. (2020). Perinatal Transmission of COVID-19 Associated SARS-CoV-2: Should We Worry?. *Clinical Infectious Diseases*.

[25] Chen S., Liao E., Cao D., Gao Y., Sun G., Shao Y. (2020). Clinical analysis of pregnant women with 2019 novel coronavirus pneumonia. *Journal of Medical Virology*.

[26] Qi H., Luo X., Zheng Y. (2020). Safe delivery for pregnancies affected by COVID-19. *BJOG: An International Journal of Obstetrics & Gynaecology*.

[27] Chen H., Guo J., Wang C. (2020). Clinical characteristics and intrauterine vertical transmission potential of COVID-19 infection in nine pregnant women: a retrospective review of medical records. *The Lancet*.

[28] Li N., Han L., Peng M. (2020). Maternal and neonatal outcomes of pregnant women with coronavirus disease 2019 (COVID-19) pneumonia: a case-control study. *Clinical Infectious Diseases*.

[29] Hantoushzadeh S., Shamshirsaz A. A., Aleyasin A. (2020). Maternal death due to COVID-19. *American Journal of Obstetrics and Gynecology*.

[30] Baud D., Greub G., Favre G. (2020). Second-trimester miscarriage in a pregnant woman with SARS-CoV-2 infection. *JAMA*.

[31] Dong Y., Chi X., Hai H. (2020). Antibodies in the breast milk of a maternal woman with COVID-19. *Emerging Microbes & Infections*.

[32] Gynecologists *Novel Coronavirus 2019 (COVID-19)*.

[33] Title N. (2020). *Royal College of Obstetricians and Gynaecologists. Coronavirus (COVID-19) infection in pregnancy*.

[34] LoMauro A., Aliverti A. (2015). Respiratory physiology of pregnancy. *Breathe*.

[35] Jamieson D. J., Honein M. A., Rasmussen S. A. (2009). H1N1 2009 influenza virus infection during pregnancy in the USA. *Lancet*.

[36] Creanga A. A., Johnson T. F., Graitcer S. B. (2010). Severity of 2009 pandemic influenza a (H1N1) virus infection in pregnant women. *Obstetrics and Gynecology*.

[37] Wong S. F., Chow K. M., Leung T. N. (2004). Pregnancy and perinatal outcomes of women with severe acute respiratory syndrome. *American Journal of Obstetrics and Gynecology*.

[38] Giakoumelou S., Wheelhouse N., Cuschieri K., Entrican G., Howie S. E. M., Horne A. W. (2015). The role of infection in miscarriage. *Human Reproduction Update*.

[39] Goodnight W. H., Soper D. E. (2005). Pneumonia in pregnancy. *Critical Care Medicine*.

[40] Ng P. C., Leung C. W., Chiu W. K., Wong S. F., Hon E. K. L. (2004). SARS in Newborns and Children. *Neonatology*.

[41] Ahmed I., Azhar A., Eltaweel N., Tan B. K. (2020). First COVID-19 maternal mortality in the UK associated with thrombotic complications. *British Journal of Haematology*.

[42] Algeri P., Stagnati V., Spazzini M. D. (2020). Considerations on COVID-19 pregnancy: a cases series during outbreak in Bergamo Province, North Italy. *The Journal of Maternal-Fetal & Neonatal Medicine*.

[43] An P., Wood B. J., Li W., Zhang M., Ye Y. (2020). Postpartum exacerbation of antenatal COVID-19 pneumonia in 3 women. *Canadian Medical Association Journal*.

[44] Buonsenso D., Raffaelli F., Tamburrini E. (2020). Clinical role of lung ultrasound for diagnosis and monitoring of COVID-19 pneumonia in pregnant women. *Ultrasound in Obstetrics & Gynecology*.

[45] Cao D., Yin H., Chen J. (2020). Clinical analysis of ten pregnant women with COVID-19 in Wuhan, China: a retrospective study. *International Journal of Infectious Diseases*.

[46] Cooke W. R., Billett A., Gleeson S. (2020). SARS-CoV-2 infection in very preterm pregnancy: experiences from two cases. *European Journal of Obstetrics, Gynecology, and Reproductive Biology*.

[47] Gong X., Song L., Li H. (2020). CT characteristics and diagnostic value of COVID-19 in pregnancy. *PLoS One*.

[48] Grimminck K., Santegoets L. A. M., Siemens F. C., Fraaij P. L. A., Reiss I. K. M., Schoenmakers S. (2020). No evidence of vertical transmission of SARS-CoV-2 after induction of labour in an immune-suppressed SARS-CoV-2-positive patient. *BMJ Case Reports*.

[49] Hu X., Gao J., Luo X. (2020). Severe acute respiratory syndrome coronavirus 2 (SARS-CoV-2) vertical transmission in neonates born to mothers with coronavirus disease 2019 (COVID-19) pneumonia. *Obstetrics and Gynecology*.

[50] Iqbal S. N., Overcash R., Mokhtari N. (2020). An uncomplicated delivery in a patient with Covid-19 in the United States. *The New England Journal of Medicine*.

[51] Kirtsman M., Diambomba Y., Poutanen S. M. (2020). Probable congenital SARS-CoV-2 infection in a neonate born to a woman with active SARS-CoV-2 infection. *Canadian Medical Association Journal*.

[52] Lang G.-J., Zhao H. (2020). Can SARS-CoV-2-infected women breastfeed after viral clearance?. *Journal of Zhejiang University. Science. B*.

[53] Lowe B., Bopp B. (2020). COVID-19 vaginal delivery - a case report. *The Australian & New Zealand Journal of Obstetrics & Gynaecology*.

[54] Lyra J., Valente R., Rosário M., Guimarães M. (2020). Cesarean section in a pregnant woman with COVID-19: first case in Portugal. *Acta Médica Portuguesa*.

[55] Panichaya P., Thaweerat W., Uthaisan J. (2020). Prolonged viral persistence in COVID-19 second trimester pregnant patient. *European Journal of Obstetrics, Gynecology, and Reproductive Biology*.

[56] Polónia-Valente R., Moucho M., Tavares M., Vilan A., Montenegro N., Rodrigues T. (2020). Vaginal delivery in a woman infected with SARS-CoV-2 - the first case reported in Portugal. *European Journal of Obstetrics, Gynecology, and Reproductive Biology*.

[57] Pulinx B., Kieffer D., Michiels I. (2020). Vertical transmission of SARS-CoV-2 infection and preterm birth. *European Journal of Clinical Microbiology & Infectious Diseases*.

[58] dos Reis H. L. B., Boldrini N. A. T., Caldas J. V. J., da Paz A. P. C., Ferrugini C. L. P., Miranda A. E. (2020). Severe coronavirus infection in pregnancy: challenging cases report. *Revista do Instituto de Medicina Tropical de São Paulo*.

[59] Richtmann R., Torloni M. R., Otani A. R. O. (2020). Fetal deaths in pregnancies with SARS-CoV-2 infection in Brazil: a case series. *Case Reports in Women's Health*.

[60] Rosen M. H., Axelrad J., Hudesman D., Rubin D. T., Chang S. (2020). Management of acute severe ulcerative colitis in a pregnant woman with COVID-19 infection: a case report and review of the literature. *Inflammatory Bowel Diseases*.

[61] Tang M. W., Nur E., Biemond B. J. (2020). Immune thrombocytopenia due toCOVID‐19 during pregnancy. *American Journal of Hematology*.

[62] Vallejo V., Ilagan J. G. (2020). A postpartum death due to coronavirus disease 2019 (COVID-19) in the United States. *Obstetrics and Gynecology*.

[63] Wu Y.-T., Li C., Zhang C.-J., Huang H.-F. (2020). Is termination of early pregnancy indicated in women with COVID-19?. *European Journal of Obstetrics, Gynecology, and Reproductive Biology*.

[64] Xia H., Zhao S., Wu Z., Luo H., Zhou C., Chen X. (2020). Emergency caesarean delivery in a patient with confirmed COVID-19 under spinal anaesthesia. *British Journal of Anaesthesia*.

[65] Xiong Y., Zhang Q., Zhao L., Shao J., Zhu W. (2020). Clinical and imaging features of COVID-19 in a neonate. *Chest*.

[66] Yang P., Wang X., Liu P. (2020). Clinical characteristics and risk assessment of newborns born to mothers with COVID-19. *Journal of Clinical Virology*.

[67] Zhang L., Dong L., Ming L. (2020). Severe acute respiratory syndrome coronavirus 2(SARS-CoV-2) infection during late pregnancy: a report of 18 patients from Wuhan, China. *BMC Pregnancy and Childbirth*.

[68] Liu W., Wang Q., Zhang Q. Coronavirus Disease 2019 (COVID-19) During Pregnancy: A Case Series.

[69] Vlachodimitropoulou Koumoutsea E., Vivanti A. J., Shehata N. (2020). COVID-19 and acute coagulopathy in pregnancy. *Journal of Thrombosis and Haemostasis*.

[70] Blauvelt C. A., Chiu C., Donovan A. L. (2020). Acute respiratory distress syndrome in a preterm pregnant patient with coronavirus disease 2019 (COVID-19). *Obstetrics and Gynecology*.

