# Supplementary material for: Pregnancy and Neonatal Outcomes in SARS-CoV-2 Infection: A Systematic Review
Source: J Pregnancy. 2020 Oct 7;2020:4592450. doi: 10.1155/2020/4592450 (PMC7542507; doi:10.1155/2020/4592450)
Supplement: Supplementary Materials — Table 1. Patient Characteristics and Symptoms in SARS-CoV-2 positive pregnancies. Table 2. Summary of Gestational Complications and Delivery Outcomes in SARS-CoV-2 positive pregnancies. [file 4592450.f1.docx]

Table 1 Patient characteristics and symptoms in SARS-CoV-2-positive pregnancies.^*,**^

| Study | Number of patients | Maternal age (years)  Mean (range) | Gestational age (weeks) | Preexisting comorbidities | | Maternal symptoms prepartum | Maternal symptoms postpartum | Imaging (CXR, CT, US) | Laboratory findings |
| --- | --- | --- | --- | --- | --- | --- | --- | --- | --- |
| Alzamora, 2020 | 1 | 41 | 33 | Respiratory insufficiency, DM | | Fever, malaise, fatigue, worsening dyspnea, tachycardia, tachypnea, metabolic acidosis |  | CT: multiple bilateral consolidations, bilateral pleural effusion | Elevated CRP, elevated ferritin, elevated D-dimer, pancytopenia, lymphopenia, decreased Hb, leukopenia, thrombocytopenia |
| Baud, 2020 | 1 | 28 | 19 | Obesity | | Fever, fatigue, myalgia, dry cough, pain with swallowing, diarrhea |  |  |  |
| Breslin, 2020 | 2 | 35.5 (33–38) | 37 (both) | Patient 1: poorly controlled type 2 DM and intrahepatic cholestasis of pregnancy  Patient 2: chronic hypertension, DM, asthma | | None | Patient 1: fever  Patient 2: cough that progressed to respiratory distress, fever, tachycardia, hypoxia, dyspnea, diaphoresis | CXR patient 2: mild pulmonary vascular congestion with no consolidation or effusion |  |
| Chen H, 2020 | 9 | 30 (26-40) | 36-39 |  | | Fever (7), cough (4), myalgia (3), sore throat (2), malaise (2), GI symptoms (1), dyspnea (1) | Fever (6) | CT: patchy GGO (8) | Elevated CRP (6), lymphopenia (5), abnormal liver enzymes (3) |
| Chen S, 2020 | 5 | 28.8 (25-31) | 38-41 |  | | Cough (2), rhinorrhea (1) | Fever (5) | CT: GGO bilaterally (3) or unilaterally (2) | Elevated CRP, decreased albumin, elevated alkaline phosphatase |
| Fan, 2020 | 2 | 34, 29 | 36, 37 |  | | Nasal congestion (2), fever (2), abdominal rash (1), sore throat (1), chills (1) |  | CT patient 1: bilateral patchy consolidation  CT patient 2: multiple patchy infiltrates on left lung | Lymphopenia (2) |
| Gidlöf, 2020 | 1 | 34 | 36 | Hypertension, proteinuria, dichorionic twin pregnancy | | Headache, hoarseness, malaise, high BP, photophobia, brisk patellar reflexes | Hypoxia | CT: typical signs of SARS-CoV-2 pneumonia | Elevated uric acid |
| Lee, 2020 | 1 | 28 | 36+2 |  | | Fever, cough, sore throat, nausea, low BP following spinal anesthesia |  | CXR: left lower/middle lobe consolidation and increased vascular marking  CT: multifocal peribronchial GGO and consolidation in left lower lobe | Elevated erythrocyte sedimentation rate (ESR), low Hb |
| Li N, 2020 | 16 | 30.9 (26-37) | 33-40 | Hypertension, hepatitis B infection, polycystic ovary syndrome | | Fever (4) | Fever (8) | Typical images of pneumonia bilaterally (10) and unilaterally (7) | Leukopenia, neutropenia, low CRP, and abnormal liver enzymes |
| Li Y, 2020 | 1 | 30 | 35 |  | | Dry cough, chills, dyspnea |  | CXR: scattered multiple patchy bilateral infiltrates | Slightly abnormal |
| Liu D, 2020 | 15 | 32 (23-40) | 12-38 | Mitral and tricuspid valve replacement (1) | | Fever (13), cough (9), sore throat (1), dyspnea (1), myalgia (3), fatigue (4), diarrhea (1) | Fever (1) | CT: GGO consolidations reported with disease progression | Elevated CRP (10), lymphocytopenia (12) |
| Liu W, 2020 | 3 | 32.7 (30–34) | 39.3 (38+ 4–40) | Patient 1: hypothyroidism and epiglottic cysts  Patient 2: none  Patient 3: gestational diabetes | | Fever (2), cough (2) |  | CT: abnormal (3) | Leukocytosis (2), neutrophilia (2), lymphocytosis (1), eosinopenia (1), thrombocytopenia (1) |
| Liu Y, 2020 | 13 | 29.7 (22-36) | 2 women less than 28 GA (25, 27). 11 in third trimester (32-36) | None | | Fever (10), cough (2), fatigue (4), dyspnea (3), sore throat (1) | MODS including ARDS (1) |  |  |
| Nie, 2020 | 33 | 30.5 (24-36) | 3 women in second trimester (17, 20, 26), 30 women in third trimester | 15 women had pre-existing chronic disorders | | Fever (21), dry cough (13), fatigue (7), dyspnea (7), asymptomatic (4)  Less common: diarrhea, muscle ache, sore throat, chest pain, stomachache | Fever (5) | CT: bilateral or unilateral pneumonia, GGO |  |
| Vlachodimitropoulou, 2020 | 2 | 40, 23 | 35+ 3, 35+ 2 | Patient 1: neutropenia, mild respiratory infections  Patient 2: not reported | | Patient 1: fever, cough, tachycardia  Patient 2: fever, cough |  | Patient 1: normal CXR  Patient 2: not reported | Patient 1: progressive thrombocytopenia, declining fibrinogen, prolonged APTT, elevated CRP, neutropenia, lymphocytopenia, elevated D-dimer  Patient 2: thrombocytopenia, prolonged APTT, transaminitis, progressive coagulopathy, elevated CRP, lymphocytopenia, elevated D-dimer |
| Wen, 2020 | 1 | 31 | 30 |  | | Mild diarrhea | Recovered and discharged prior to delivery | CT: patchy consolidation and GGO |  |
| Yu, 2020 | 7 | 32 (29-34) | 39+ 1 (37-41+ 2) | Hypothyroidism (1), polycystic ovary syndrome (1) | | Fever (6), cough (1), dyspnea (1), diarrhea (1) |  | CT: bilateral pneumonia (6), unilateral pneumonia (1) | Elevated CRP (7), neutrophilia (5), lymphopenia (5), low platelets (2), elevated D-dimer (7), abnormal liver enzymes (2) |
| Zhu, 2020 | 9 | 30 (25-35) | 31-39 |  | | Fever (8), cough (4), diarrhea (1), sore throat (1), cholecystitis (1) |  | CT: typical of viral pneumonia. Lesions merged into strips as disease progressed |  |
| Ahmed, 2020 [41] | 1 | 29 | 26 | Obesity, DM, asthma, renal tubular acidosis | | Fever | Pulmonary embolism | CT: right lower lobar pulmonary embolism, extensive bilateral GGO and patchy solid consolidation |  |
| Algeri, 2020 [42] | 5 | — | 36 |  | | Fever (3), cough (1), asymptomatic (1) | Fever (1), cough (1), GI symptoms (1), hypoxia (2) | CT: GGO and consolidation (1), interstitial pneumonia (2), cardiomegaly (1) | Thrombocytopenia (3), leukopenia (2), abnormal liver enzymes (1), elevated CRP (1), long QT syndrome (1) |
| An, 2020 [43] | 3 | 29.7 (25–33) | 38.3 (38–39) |  | | Fever (3), cough (2), fatigue (1), dyspnea (2), bilateral lower leg edema (1), GI symptoms (1) | Cough (3), dyspnea (2), hypoxia (3) | CT: GGO (3) | Elevated CRP (3), neutrophilia (1), leukocytosis (1) |
| Blauvelt, 2020 [44 ] | 1 | 34 | 28 | Obesity, asthma | | Fever, cough, fatigue, myalgia, dyspnea | UTI | CT: bilateral opacities | Elevated CRP, elevated D-dimer, elevated lactate, lymphopenia |
| Buonsenso, 2020[44] | 2 | 40 (38–42) | 36.5 (35–38) |  | | Cough (2), dyspnea |  | US: diffuse interstitial lung disease |  |
| Cao, 2020 [45] | 10 | 30.3 (29–35) | 37.4 (33–40) |  | | Fever (2), cough (1), fatigue (1) | Fever (5) | CT: single lobe lesions (4), bilateral lobe lesions (6), GGO (4), pleural effusions (5) | Elevated CRP (6), lymphopenia (6) |
| Cooke, 2020 [46] | 2 | 33.5 (28–39) | 28, 28 | DM (1), obesity (1) | | Fever (1), dyspnea (1), cough (2), GI symptoms (1), | Hypoxia (1) | CT: bilateral infiltrates (1), bibasal pulmonary infiltrates (1) |  |
| Dong, 2020 | 1 | 33 | 38 |  | | Cough, dyspnea |  | CT: patchy GGO | Neutrophilia, abnormal liver enzymes |
| Gong, 2020 [47] | 10 | 39.4 | Median: 37 |  | | Fever (8), cough (5), GI symptoms (2), nasal congestion (1), asymptomatic (1) |  | CT: GGO (10), consolidation (6), bilateral pleural effusions (6) | Elevated CRP (7), neutrophilia (10), elevated D-dimer (8), decreased protein (6) |
| Grimminck, 2020 [48] | 1 | 31 | 38 | Hypertension, SLE | | Cough, dyspnea |  |  |  |
| Hantoushzadeh, 2020 | 9 | 33.3 (25–45) | 30.3 (24–38 3/7) | Obesity (3), subclinical hypothyroidism (1), gestational diabetes (1) | | Fever (7), cough (7), dyspnea (4), myalgia (3) |  | CT: GGO (6) | Elevated CRP (7), lymphopenia (1), pancytopenia (1), elevated liver enzymes (1) |
| Hu 2020 [49] | 7 | 33.14 | 38.71 |  | | Fever (4), cough (2), GI symptoms (1), asymptomatic (1) |  |  |  |
| Iqbal, 2020 [50] | 1 | 34 | 39 |  | | Fever, cough, myalgia |  | CT: reticular interstitial opacities | Lymphopenia |
| Kirtsman, 2020 [51] | 1 | 40 | 35 | Familial neutropenia | |  |  |  | Elevated D-dimer, thrombocytopenia, elevated fibrinogen |
| Lang, 2020 [52] | 1 | 30 | 35 |  | | Cough |  |  | Elevated CRP, neutrophilia |
| Lowe, 2020 [53] | 1 | 31 | 40 |  | | Upper respiratory symptoms |  |  |  |
| Lyra, 2020 [54] | 1 | 35 | 39+ 6 |  | | Cough |  |  |  |
| Panichaya, 2020 [55] | 1 | 43 | 18 |  | | Fever, chest discomfort |  |  |  |
| Polónia-Valente, 2020 [56] | 1 | 31 | 38 |  | | Dry cough |  |  |  |
| Pulinx, 2020 [57] | 1 | 30 | 22 |  | | Fever, rhinitis |  |  | Elevated CRP |
| Reis, 2020 [58] | 3 | 29 (25–34) | 28+ 6. 31, 1 day postpartum |  | | Fever (3), dyspnea (2), abdominal pain (2), cough (2), myalgia (1) |  |  | Elevated CRP (3) |
| Richtmann, 2020 [59] | 5 | 32.8 (24–40) | 28 (21 (1/7)–38 (3/7)) | Obesity (2) | | Fever (3), cough (2), dyspnea (1), rhinorrhea (3), myalgia (1), abdominal pain (1) |  |  | Neutrophilia (5), lymphocytosis (5) |
| Rosen, 2020 [60] | 1 | 26 | Could not determine | Ulcerative pancolitis | | Abdominal pain, diarrhea, hematochezia |  |  | Elevated CRP |
| Tang, 2020 [61] | 1 |  | 41 |  | | Sore throat |  | CT: GGO | Thrombocytopenia |
| Vallejo, 2020 [62] | 1 | 36 | 37 | Obesity, appendectomy, abdominoplasty, spontaneous abortion | | Fever, dyspnea, dry cough, sore throat, tachypnea | Septic shock | CXR: moderate air space opacities and focal consolidation | Leukocytosis, elevated ESR, elevated D-dimer, abnormal liver enzymes, increased troponin |
| Wu, 2020 [63] | 23 | 25 (21–37) | 6-40 | Hypothyroidism (2), hepatitis B (2) | | Fever (4), cough (5), nasal congestion (1) |  | CT: GGO (20), patchy consolidation (18), multiple lobe involvement (14) |  |
| Xia, 2020 [64] | 1 | 27 | 36 |  | | Fever |  | CT: GGO, multiple bilateral patchy lung consolidation | Elevated CRP, neutrophilia, elevated Hb |
| Xiong, 2020 [65] | 1 | 34 | 40 |  | | Fever |  | CT: bilateral GGO | Elevated CRP, lymphopenia |
| Yang, 2020 [66] | 7 |  | 36+ 8 (36–38+ 2) |  | | Fever (5), cough (1), diarrhea (1), abdominal pain (1) |  | CT: viral pneumonia (6) | Increased PCT (1), increased D-dimer, abnormal liver enzymes (3) |
| Zhang, 2020 [67] | 18 | 29.11 (24–34) | 38.4 (35.7–41) |  | | Fever (5), cough (3), sore throat (1), fatigue (1), dyspnea (2), diarrhea (1), rhinorrhea and nasal congestion (1) |  | CT: unilateral pneumonia (9), bilateral pneumonia (6), pneumonia with pleural effusion (2) | Elevated CRP (10), neutrophilia (14), leukocytosis (7), lymphopenia (8), decreased Hb (4), increased PCT (5), increased LDH (5), abnormal liver enzymes (5) |
| Total $n$ | | | | | 245 | | | | |

^*^The number of newborns is different from the number of pregnancies due to certain studies not following the pregnancy to birth and due to twin pregnancies. ^**^Blank cells in Table 1 denote information that was not reported in the publication.

Table 2 Summary of gestational complications and delivery outcomes in SARS-CoV-2-positive pregnancies.^*,**^.

| Study | Number of newborns | C-section | Vaginal delivery | 1 min Apgar scores | 5 min Apgar scores | Neonatal complications | Newborn and tissue testing | Breastfeeding transmission |
| --- | --- | --- | --- | --- | --- | --- | --- | --- |
| Alzamora, 2020 | 1 | 1 |  | 6 | 8 |  | Neonate positive testing at hour 16 of life | |
| Baud, 2020 | 3 | 2 | 1 |  |  | Stillbirth | Placenta tested positive |  |
|  |  |  |  |  |  |  | Negative neonate testing (2) |  |
| Breslin, 2020 | 2 | 2 |  |  |  | Preterm birth (4) |  |  |
| Chen H., 2020 | 9 | 9 |  |  |  |  |  |  |
|  |  |  |  |  |  |  |  |  |
| Chen S., 2020 | 5 | 2 | 3 | 10 | 10 |  |  |  |
|  |  |  |  | Average of $n = 5$ | Average of $n = 5$ |  |  |  |
| Fan, 2020 | 2 | 2 |  | 9 | 10 | Neonatal CT shows diffuse haziness in both lung fields (1) | Negative neonate testing (2) | Negative breast milk testing |
|  |  |  |  |  |  |  | Negative placental samples (2) |  |
|  |  |  |  |  |  |  | Negative amniotic fluid testing (2) |  |
|  |  |  |  |  |  |  | Negative maternal vaginal swab (2) |  |
|  |  |  |  |  |  |  | Negative umbilical cord blood (2) |  |
| Gidlöf, 2020 [13] | 2 | 1 |  | 9 | 10 | Neonatal respiratory distress (1), resolved after CPAP | |  |
|  |  |  |  | 9 | 10 |  |  |  |
| Lee, 2020 |  | 1 |  | 9 | 10 |  | Negative neonate testing |  |
| Li N., 2020 | 16 | 14 | 2 |  |  | Preterm birth (5) |  |  |
| Li Y., 2020 | 1 | 1 |  |  |  |  |  |  |
| Liu D., 2020 | 11 | 10 | 1 | 8 | 9 |  |  |  |
|  |  |  |  | Average of $n = 11$ | Average of $n = 1$1 |  |  |  |
| Liu W., 2020 [68] |  | 2 | 1 |  |  |  |  |  |
| Liu Y., 2020 | 11 | 10 | 1 |  | 10 (9) | Stillbirth |  |  |
|  |  |  |  |  |  | Preterm birth (6) |  |  |
| Nie, 2020 | 28 | 22 | 5 | Range of $n = 27$: 8-10 | Range of $n = 27$: 9-10 | Preterm birth (19) | Negative neonate testing (26) |  |
|  |  |  |  |  |  | Neonatal ARDS (1) | Positive neonate testing (1) |  |
| Vlachodimitropoulou, 2020 [69] | 2 | 2 |  | 9 | 10 |  |  |  |
|  |  |  |  | 4 | 2 |  |  |  |
| Wen, 2020 | NA | NA |  |  |  |  |  |  |
| Yu, 2020 | 7 | 7 |  | 8.5 | 9.5 |  | Negative neonate testing (2) |  |
|  |  |  |  | Average of $n = 7$ | Average of $n = 7$ |  | Positive neonate testing (1) |  |
| Zhu, 2020 | 10 | 7 | 2 | 8.6 | 9.4 | Preterm birth (6) | Negative neonate testing (9) |  |
|  |  |  |  | Average of $n = 10$ | Average of $n = 10$ | Neonatal shortness of breath (6) | |  |
|  |  |  |  |  |  | Neonatal death (1) due to refractory shock and multiorgan failure on day 8 of life | | |
| Ahmed, 2020 [41] | 1 | 1 |  |  |  | Preterm birth | Negative neonate testing |  |
|  |  |  |  |  |  |  |  |  |
| Algeri, 2020 [42] | 1 |  | 1 |  |  | Preterm birth |  |  |
|  | 5 | 4 |  |  |  |  |  |  |
|  |  |  |  |  |  |  |  |  |
| An, 2020 [43] | 3 | 3 |  |  |  |  | Negative neonate testing (3) |  |
| Blauvelt, 2020 [70] | 1 | 1 |  | 4 | 8 | Preterm birth | Negative neonate testing |  |
|  |  |  |  |  |  | Neonatal CT: diffuse lung bilateral granular and hazy opacities | |  |
| Buonsenso, 2020 [44] |  | 1 |  | 9 | 10 | Preterm birth | Placenta and umbilical blood were negative | Negative breast milk testing |
|  |  | 1 |  | 8 | 9 |  | Positive neonate testing on DOL 15 |  |
|  |  |  |  |  |  |  | Placenta and umbilical blood were positive | Positive breast milk testing (3/5) |
|  |  |  |  |  |  |  | Negative neonate testing on DOL 18 | |
| Cao, 2020 [45] | 11 | 6 (elective C-section) | 2 | 8 ($n = 4$) | 10 ($n = 10$) | Preterm birth (4) |  |  |
|  |  | 2 (emergency C-section) | | 9 ($n = 6$) |  |  |  |  |
| Cooke, 2020 [46] | 2 | 2 |  | 6 | 8 | Preterm birth (2) | Negative neonate testing (2) |  |
|  |  |  |  | 1 | 3 |  |  |  |
| Dong, 2020 | 1 |  | 1 | 9 | 10 |  | Placental transmission of SARS-CoV-2 IgG antibody | Breast milk positive for SARS-CoV-2 IgG and IgA (but negative for SARS-CoV-2) |
| Gong, 2020 [47] | NA |  |  |  |  |  |  |  |
| Grimminck, 2020 [48] | 1 |  | 1 | 9 | 10 |  | Vaginal, maternal urine, maternal and fetal placentas, neonatal oropharynx: all negative | |
|  | 6 | 6 | 1 | 6 | 7 | Preterm birth (4) |  |  |
| Hantoushzadeh, 2020 |  |  |  | 7.8 | 9.2 | Neonatal pneumonia |  |  |
|  |  |  |  | Average of $n = 6$ | Average of $n = 6$ | Neonatal lymphopenia |  |  |
|  |  |  |  |  |  | Neonatal death (twins) |  |  |
| Hu, 2020 [49] | 7 | 6 | 1 | 7.85 | 8.85 |  | Positive neonate testing | All neonates formula-fed: 6/7 negative for SARS-CoV-2 |
|  |  |  |  | Average of $n = 7$ | Average of $n = 7$ |  |  |  |
| Iqbal, 2020 [50] | 1 |  | 1 | 8 | 9 |  | No evidence of neonatal or intra-amniotic infection | Breast milk given and no evidence of neonatal infection |
| Kirtsman, 2020 [51] | 1 | 1 |  | 9 | 9 | Preterm birth | Negative neonate testing |  |
| Lang, 2020 [52] | 1 | 1 |  | 9 | 10 |  | Maternal urine, stool, cord blood, amniotic fluid, placenta, baby’s oropharyngeal swab: all negative | Negative breast milk testing |
| Lowe, 2020 [53] | 1 |  |  | 9 | 9 |  | Negative neonate testing |  |
| Lyra, 2020 [54] | 1 | 1 |  | 8 | 9 |  | Negative neonate testing |  |
| Panichaya, 2020 [55] | 1 |  | 1 |  |  |  | Negative neonate testing |  |
| Polónia-Valente, 2020 [56] | 1 |  | 1 | 9 | 10 |  | Negative neonate testing |  |
| Pulinx, 2020 [57] | 2 |  | 1 |  |  | Preterm birth (2) | Positive placental tissue and amniotic fluid testing | |
| Reis, 2020 [58] | 3 | 3 |  | 8, 8, 6 | 9, 9, 7 | Preterm birth (2) |  |  |
| Richtmann, 2020 [59] |  | 2 (the rest not reported) | |  |  |  |  |  |
| Rosen, 2020 [60] |  |  |  |  |  |  |  |  |
| Tang, 2020 [61] | 1 | 1 |  |  |  |  |  |  |
| Vallejo, 2020 [62] | 1 | 1 |  | 5 | 9 |  |  |  |
| Wu, 2020 [63] | 21 | 18 | 2 | NA | 9-10 for all neonates | Other: neonatal jaundice (1) | Negative neonate testing (21) |  |
|  |  |  |  |  |  |  |  |  |
| Xia, 2020 [64] | 1 | 1 | 0 | 9 | 10 |  |  |  |
| Xiong, 2020 [65] | 1 | 1 |  | 8 | 9 |  | Positive neonate testing on hour 36 of life | |
| Yang, 2020 [66] | 7 | 7 | 0 | 8 ($n = 5$), 9 ($n = 2$) | 9 ($n = 5$), 10 ($n = 2$) | Hypoglycemia (1) | Amniotic fluid, umbilical cord blood, and throat swabs tested negative | |
|  |  |  |  |  |  | Preterm birth (2) |  |  |
| Zhang, 2020 [67] | 18 | 17 | 1 |  |  | Preterm birth (3) | Negative neonate testing (18) | Negative breast milk testing |
|  |  |  |  |  |  | Other: mild asphyxia |  |  |
|  |  |  |  |  |  | Other: GI bleed |  |  |
|  |  |  |  |  |  | Other: necrotizing enteritis |  |  |
|  |  |  |  |  |  | Other: hyperbilirubinemia |  |  |
|  |  |  |  |  |  | Other: diarrhea |  |  |
| Total | 201 newborns | 179 C-sections | 30 vaginal deliveries | Average 6.49 | Average 8.98 | 30 studies with reported neonatal complications:  71 cases of preterm birth  5 cases of stillbirth and neonatal death  15 other problems | | |

^*^The number of newborns is different from the number of pregnancies due to certain studies not following the pregnancy to birth and due to twin pregnancies. ^**^Blank cells in Table 2 denote information that was not reported in the publication.
